# The “Lipid Accumulation Product” Is Associated with 2-Hour Postload Glucose Outcomes in Overweight/Obese Subjects with Nondiabetic Fasting Glucose

**DOI:** 10.1155/2015/836941

**Published:** 2015-02-22

**Authors:** Alexis Elias Malavazos, Emanuele Cereda, Federica Ermetici, Riccardo Caccialanza, Silvia Briganti, Mariangela Rondanelli, Lelio Morricone

**Affiliations:** ^1^Nutrition and Dietetics Service, Fondazione IRCCS Policlinico San Matteo, Viale Golgi 19, 27100 Pavia, Italy; ^2^U.O. di Diabetologia e Malattie Metaboliche, IRCCS Policlinico San Donato, Via Morandi 30, San Donato Milanese, 20097 Milano, Italy; ^3^Ambulatorio di Dietologia, Dipartimento di Scienze Sanitarie Applicate e Psicocomportamentali, Sezione di Nutrizione, Azienda di Servizi alla Persona di Pavia, Università degli Studi di Pavia, via Emilia No. 12, 27100 Pavia, Italy

## Abstract

“Lipid accumulation product” (LAP) is a continuous variable based on waist circumference and triglyceride concentration previously associated with insulin resistance. We investigated the accuracy of LAP in identifying oral glucose tolerance test (OGTT) abnormalities and compared it to the homeostasis model assessment of insulin resistance (HOMA-IR) in a population of overweight/obese outpatients presenting with nondiabetic fasting glucose. We studied 381 (male: 23%) adult (age: 18–70 years) overweight/obese Caucasians (body mass index: 36.9 ± 5.4 Kg/m^2^) having fasting plasma glucose < 7.0 mmol/L. OGTT was used to diagnose unknown glucose tolerance abnormalities: impaired glucose tolerance (IGT) and type-2 diabetes mellitus (T2-DM). According to OGTT 92, subjects had an IGT and 33 were diagnosed T2-DM. Logistic regression analysis detected a significant association for both LAP and HOMA-IR with single (IGT and T2-DM) and composite (IGT + T2-DM) abnormal glucose tolerance conditions. However, while the association with diabetes was similar between LAP and HOMA-IR, the relationship with IGT and composite outcomes by models including LAP was significantly superior to those including HOMA-IR (*P* = 0.006 and *P* = 0.007, resp.). LAP seems to be an accurate index, performing better than HOMA-IR, for identifying 2-hour postload OGTT outcomes in overweight/obese patients with nondiabetic fasting glucose.

## 1. Introduction

Impaired glucose tolerance to overt diabetes is substantially regarded as an obesity-related complication [1]. The pathophysiological role of excessive visceral adiposity in the decline of pancreatic *β*-cell function is well accepted [2, 3]. Alternatively to body mass index (BMI, describing weight overaccumulation), a new index describing central lipid overaccumulation, the “lipid accumulation product” (LAP), has been recently proposed for identifying adults with insulin resistance, elevated fasting glucose, and diabetes [4–6]. LAP is a continuous variable based on waist circumference (WC) and triglyceride (TG) concentration, two elements denoting visceral adiposity [3, 4].

A pathologic glucose tolerance and a degree of hyperglycemia sufficient to cause functional changes in various target tissues, but without clinical symptoms, may be present for a long period of time before diabetes is detected [1]. In the identification of subjects with glucose metabolism abnormalities but presenting with normal or mildly increased fasting glucose, although not recommended for routine clinical use, the use of a 2-hour postload glucose of an oral glucose tolerance test (OGTT) is an accepted procedure [1].

As LAP likely reflects insulin resistance, we aimed to investigate the accuracy of LAP in identifying OGTT abnormalities and to compare it to a widely used index of insulin sensitivity, the homeostasis model assessment of insulin resistance (HOMA-IR) [7]. However, although the identification of normal-weight subjects with alterations in glucose tolerance is also relevant, in the present study we decided to focus only on a population of overweight/obese outpatients presenting with nondiabetic fasting glucose. We decided to include only this kind of patients in order to better investigate the discriminatory power of this new index in a population already characterized by consistent truncal adipose tissue accumulation and more at risk of developing metabolic complications. Furthermore, silent glucose tolerance abnormalities are more likely to be not diagnosed in patient with normal fasting glucose when they are not tested by means of specific procedures. Finally, the evaluation of overweight/obese outpatients reasonably reflects everyday clinical practice.

## 2. Materials and Methods

The study protocol was approved by the local ethics committees. All procedures followed were in accordance with the ethical standards of the responsible committee on human experimentation (institutional and national) and with the Helsinki Declaration of 1975, as revised in 2008. Informed consent was obtained from all patients for being included in the study. We studied 381 (male: 23%) overweight/obese Caucasians (BMI mean ± SD: 36.9 ± 5.4 Kg/m^2^; range: 27.3–55.8 Kg/m^2^; age: 41.3 ± 12.5 years) attending outpatient clinics for weight concern. Subjects were eligible if they were apparently healthy at physical examination, aged from 18 to 70 years, had fasting plasma glucose <7.0 mmol/L, and provided informed consent. Exclusion criteria were established diabetes or presence of any other endocrine disorder, use of insulin or any other hypoglycemic agent, use of lipid-lowering medications, TG ≥ 5.6 mmol/L [4, 5], pregnancy, alcohol abuse, and adherence to any weight reducing or low carbohydrate diet in the last 6 months.

Study protocol included physical examination; anthropometric measurements (weight, height, WC, and BMI) according to standard procedures [8]; biochemical assessment (glucose, insulin, and TG) of blood samples in fasted state (8 to 12 hours); glucose tolerance evaluation by two-hour postload (75 g) blood glucose of an oral glucose tolerance test (2 h-PG) [1]. Accordingly, patients were assigned to the following categories of glucose tolerance: <7.8 mmol/L, normal glucose tolerance; ≥7.8 and <11.1 mmol/L, impaired glucose tolerance (IGT); ≥11.1 mmol/L, type-2 diabetes mellitus (T2-DM). Insulin resistance was estimated by the homeostasis model assessment (HOMA-IR) of insulin resistance [7]. LAP was obtained using the formula proposed by Kahn [4]: for men = (WC [cm] − 65) × (TG [mmol/L]); for women = (WC [cm] − 58) × (TG [mmol/L]). Patients were then stratified by sex-specific tertiles of WC, LAP, and HOMA-IR distribution.

### 2.1. Statistical Analysis

Data were presented as mean and standard deviation (SD) or counts and percentage, as appropriate.

Group comparisons were performed using chi-square test (categorical variables) and ANOVA or Kruskal-Wallis test (continuous variables). Adjustment for multiple comparison by Bonferroni's procedure was considered accordingly. Logistic regression analysis adjusted for age (continuous) and smoking (current versus former smoker/nonsmoker) was used to evaluate the relationship between abnormal glucose tolerance conditions (IGT, T2-DM, and composite IGT + T2-DM) and tertiles of WC, LAP, and HOMA-IR. Therefore, the power of model's predicted values to correctly classify positive cases was also quantified by the area under the receiver operating characteristic curve (AUC;* c*-statistic). With respect to this, the closer to 1, the better the model performance [9]. Finally, AUCs for WC, LAP, and HOMA-IR were compared using the method proposed by DeLong et al. [10].

All statistical analyses were performed using the software MEDCALC for Windows, Version 11.3.0.0 (MedCalc Software, Mariakerke, Belgium). The level of significance was set at the two-tailed* P* value < 0.05.

## 3. Results

In our population sex-specific cut points of LAP tertiles (cm·mmol/L) were males, 65.8 and 103.4 (range: 21.6–247.9); females, 44.7 and 81.5 (range: 11.6–215.4). Cut points of HOMA-IR tertiles were males, 2.4 and 3.7 (range: 0.6–13.2); females, 2.2 and 3.7 (range: 0.4–13.4). Cut points of WC tertiles were males, 110 and 123 (range: 85–150); females, 96.5 and 109 (range: 80.5–139). Smoking habit was unrelated to WC, LAP, and HOMA-IR tertiles.

According to OGTT 92 subjects (24.1%) had an IGT and 33 (8.7%) were diagnosed with T2-DM. In those presenting with fasting glucose ≥ 5.6 mmol/L (*N* = 102), 22 subjects were diagnosed with T2-DM and 33 showed an IGT, while in the subgroup of patients presenting fasting glucose ≥ 6.1 mmol/L (*N* = 38) 15 and 8 cases of T2-DM and IGT were found, respectively.

**Figure 1:**
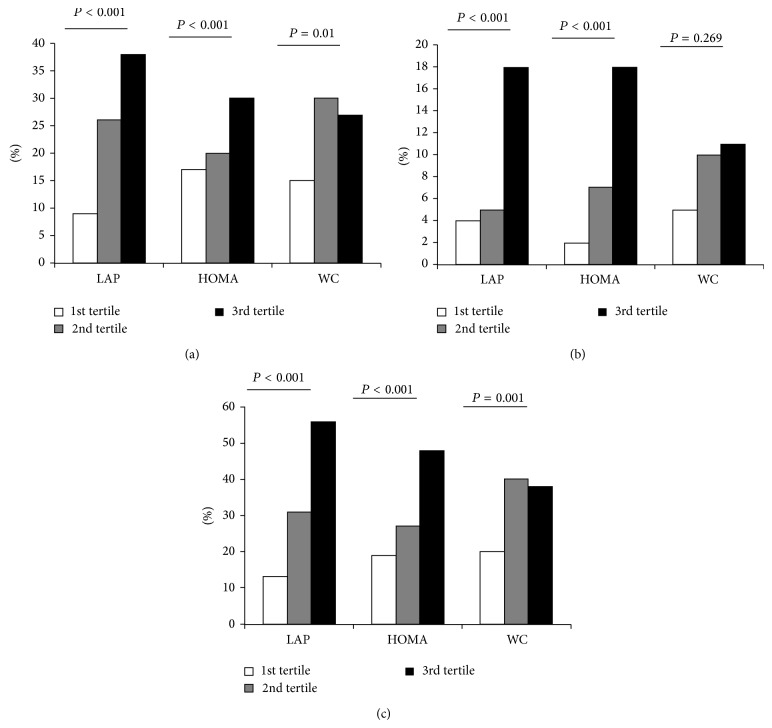
Prevalence of abnormalities in glucose metabolism by 2-hour postload glucose of an oral glucose tolerance test (*Plot* (a), IGT;* Plot* (b), T2-DM;* Plot* (c), composite glucose tolerance conditions (IGT + T2-DM)) according to tertiles of waist circumference, LAP, and HOMA-IR in the whole study population.

According to tertiles of distribution, LAP and HOMA-IR were significantly associated with variables describing glucose metabolism in both genders (Tables 1 and 2), with exception of fasting glucose and LAP in male patients. Conversely, WC was not associated with 2h-PG on a continuous scale. When looking at the frequency of abnormal glucose tolerance conditions, we found that LAP was more strongly associated with 2h-PG outcomes (Figure 1), while WC showed significant limitations. All these findings were particularly evident in age and smoking-adjusted logistic regression analysis (Table 3). A significant association for both LAP and HOMA-IR with single (IGT and T2-DM) and composite (IGT + T2-DM) abnormal glucose tolerance conditions was observed. However, while the association with diabetes was similar between LAP and HOMA-IR, the relationship with IGT and composite abnormal glucose tolerance conditions by models including LAP was significantly superior to those including HOMA-IR. Besides, WC was not inferior to HOMA-IR in predicting most glucose metabolism abnormalities with exception of T2-DM.

Therefore, the same models were refitted to patients with fasting glucose < 5.6 mmol/L (*N* = 279). In this subset of patients, LAP was confirmed to be a stronger correlate of glucose tolerance abnormalities than HOMA-IR: for composite outcomes, OR_LAP_ = 2.76 [95% CI, 1.88–4.07] (*P* < 0.001) versus OR_HOMA - IR_ = 1.69 [95% CI, 1.18–2.42] (*P* < 0.001) and AUC_LAP model_ = 0.74 [95% CI, 0.68–0.79] versus AUC_HOMA-IR model_ = 0.64 [95% CI, 0.58–0.70] (for comparison,* P* = 0.025); for IGT, OR_LAP_ = 2.45 [95% CI, 1.65–3.64] (*P* < 0.001) versus OR_HOMA - IR_ = 1.60 [95% CI, 1.10–2.32] (*P* = 0.013) and AUC_LAP model_ = 0.71 [95% CI, 0.65–0.76] versus AUC_HOMA-IR model_ = 0.61 [95% CI, 0.55–0.67] (for comparison,* P* = 0.041); for T2-DM, OR_LAP_ = 3.02 [95% CI, 1.09–8.35] (*P* = 0.033) versus OR_HOMA - IR_ = 1.77 [95% CI, 0.76–4.13] (*P* = 0.188) and AUC_LAP model_ = 0.80 [95% CI, 0.72–0.85] versus AUC_HOMA-IR model_ = 0.76 [95% CI, 0.70–0.81] (for comparison,* P* = 0.640).

Finally, BMI (by tertiles of its distribution in the whole population) was mildly associated with composite 2-hour OGTT outcomes (OR = 1.58 [95% CI, 1.19–2.11],* P* = 0.002) and IGT (OR = 1.60 [95% CI, 1.18–2.19],* P* = 0.003) and did not associate with T2-DM (OR = 1.18 [95% CI, 0.74–1.88],* P* = 0.496).

## 4. Discussion

In the present study, we have demonstrated that LAP is an accurate and reliable index for identifying not only overall 2-hour OGTT outcomes but also the prediabetic state of hyperglycemia, namely, IGT, in overweight/obese patients with nondiabetic fasting glucose. Findings were more consistent in female than male patients, although this is likely related to limitations in statistical power.

IGT is strictly associated with insulin resistance and our results confirmed previous evidence that LAP is a significant correlate of insulin resistance [4–6]. Since WC and TG are included among the criteria of metabolic syndrome (or insulin-resistant dyslipidemic syndrome), their role in suggesting insulin resistance is well accepted [3, 6, 11]. However, WC alone appeared to be less valid parameters of glucose metabolism abnormalities as it applies to overweight/obese patients. To the best of our knowledge, this is the first comparison between LAP and WC. Moreover, we have demonstrated for the first time that LAP is superior to HOMA-IR in identifying different degrees of pathological glucose tolerance in this patient population, even in those subjects with normal fasting glucose (<5.6 mmol/L). With respect to this, it is worth mentioning that subclinical organ damage associated with abnormal glucose tolerance starts before the onset of overt diabetes [12].

Progressive lipid accumulation, particularly in the abdominal region, is characterized by an increase of insulin resistance. As it is structured, it is reasonable to argue that LAP is able to reflect both visceral fat mass deposition and an increased lipolytic activity within this adipose tissue compartment [2, 3]. The accuracy of LAP in identifying glucose metabolism abnormalities has been already suggested [5]. In the NHANES III (Third National Health and Nutrition Examination Survey) population LAP showed a higher association with T2-DM than BMI [5]. Particularly, subjects in the upper sex-specific quartiles of LAP demonstrated over twice the likelihood of BMI quartiles of having diabetes. However, the cut points for the upper quartiles were 28.9 kg/m^2^ and 29.6 kg/m^2^ in men and women, respectively, and the role of LAP in obese subjects was not addressed [5]. Similar findings have been confirmed by a large study in young Korean women [13]. However, also in this study most 2h-PG abnormalities were in the upper quintile of LAP distribution in which the mean BMI was 25.5 kg/m^2^. In our study, we included only overweight/obese patients who are characterized by consistent truncal adipose tissue accumulation and more at risk of developing metabolic complications. Accordingly, LAP appeared to be a good correlate of glucose tolerance abnormalities also in the presence of considerable weight excess, a condition in which the relationship between fat mass and body weight may be no longer linear [3]. On the other hand, WC alone was a less performant index. Moreover, LAP appeared to identify glucose metabolism abnormalities more accurately than HOMA-IR, a commonly assessed and widely used index of glucose tolerance. This is even more interesting as the object of the present investigation was the association with latent glucose metabolism abnormalities. In NHANES III population, the diagnosis of diabetes was defined by report of a physician or the use of specific medications or by fasting glucose ≥ 11.1 mmol/L. Latent form of diabetes may have passed unrecognized and other degrees of abnormal glucose tolerance have not been investigated. Also these considerations may support the value of LAP and its use in clinical practice.

Indeed, the present study was also a cautious attempt of validating inexpensive research tools for the screening of glucose tolerance abnormalities. Waist circumference and TG concentrations measurements are low-cost procedures, accessible to all general practitioners. On the other hand, OGTT is not of clinical routine use and the assessment of fasting insulin, also for HOMA-IR calculation, is not recommended by international guidelines.

Some limitations to our study should be acknowledged and discussed. Although the literature on the use of OGTT in the assessment of glucose tolerance abnormalities is extensive, it should be recognized that dynamic parameters of insulin sensitivity could provide valuable and more appropriate information [14]. The setting of recruitment of our study population may be considered the main limitations of the present study. However, it could also be considered a point of strength as the outpatient setting and the inclusion of overweight/obese patients more likely reflect the daily clinical practice. A further limitation is also the size of study population, particularly the number of male patients that did not allow providing a clinical threshold value for LAP beyond which OGTT should be recommended. Finally, an unmeasured confounder of our study was physical activity but in the presence of overweight/obesity this factor is less likely to be a source of bias.

The association between LAP and glucose metabolism abnormalities should be probably investigated by means of prospective investigations. With respect to this, large population studies (cross-sectional and cohort) would allow proposing cut-off values to be used in clinical practice for risk screening.

## Figures and Tables

**Table 1:** Features of male patients according to tertiles of waist circumference, LAP, and HOMA-IR.

	Age (years)^a^	BMI (kg/m^2^)^a^	Waist (cm)^a^	LAP (cm × mmol/L)^b^	Glucose (mmol/L)^b^	Insulin (*μ*U/dL)^b^	HOMA-IR^b^	2h-PG (mmol/L)^b^
Overall (*N* = 87)	45.6 (12.9)	36.7 (6.1)	116.4 (13.2)	85.8 [54.6–120.3]	5.3 (0.7)	16.4 (9.2)	3.9 (2.4)	6.3 [5.6–8.1]
Waist tertiles								
I (*N* = 30)	45.3 (13.0)	31.2 (2.7)	102.6 (15.3)	55.2 [41.3–82.7]^†^	5.1 (0.7)	12.8 (8.5)^‡^	3.0 (2.4)^‡^	6.3 [5.6–8.2]
II (*N* = 26)	46.6 (14.2)	35.9 (3.0)	115.3 (3.3)	89.8 [71.0–120.5]	5.5 (0.7)	15.8 (7.0)	3.9 (2.1)	6.3 [5.7–8.6]
III (*N* = 31)	45.0 (12.3)	42.8 (5.0)	130.8 (7.8)	103.3 [76.7–132.1]	5.2 (0.7)	20.3 (10.2)	4.7 (2.4)	6.0 [5.0–7.8]
*P* value^*^	0.895	<0.001^§^	<0.001^§^	0.002	0.179	0.006	0.017	0.428
LAP tertiles								
I (*N* = 30)	46.0 (13.8)	33.0 (4.2)	107.9 (11.3)^†^	49.9 [40.2–56.1]	5.1 (0.7)	12.2 (7.0)^‡^	2.8 (1.8)^‡^	6.0 [4.9–7.0]^‡^
II (*N* = 29)	47.6 (13.2)	36.5 (4.8)	118.0 (9.3)	86.8 [75.5–96.5]	5.3 (0.7)	16.0 (6.8)	3.9 (2.0)	6.1 [5.7–7.9]
III (*N* = 28)	43.2 (12.1)	41.1 (6.6)	124.0 (13.7)	131.0 [121.0–174.3]	5.3 (0.8)	21.2 (11.4)	5.0 (2.9)	6.4 [5.9–8.6]
*P* value^*^	0.451	<0.001^§^	<0.001	<0.001^§^	0.525	<0.001	0.003	0.049
HOMA tertiles								
I (*N* = 27)	45.8 (13.0)	33.9 (5.4)^‡^	109.6 (11.3)	54.6 [41.3–89.5]^‡^	4.8 (0.6)^†^	7.7 (2.8)	1.6 (0.5)	6.1 [4.9–7.1]
II (*N* = 28)	45.7 (14.7)	36.6 (5.1)	116.7 (13.0)	82.0 [55.1–121.1]	5.4 (0.5)	13.5 (1.9)	3.2 (0.4)	5.7 [4.9–7.2]
III (*N* = 32)	44.6 (11.9)	39.4 (6.7)	121.8 (13.3)	101.3 [69.7–131.5]	5.5 (0.8)	25.8 (27.8)	6.3 (2.2)	6.4 [6.0–8.8]^†^
*P* value^*^	0.927	0.003	0.002	0.001	0.002	<0.001^§^	<0.001^§^	0.010

Data are reported as mean (standard deviation)^a^ or median [interquartile range, 25th–75th percentile]^b^ or percentage. Percentages are calculated within groups.

BMI, body mass index; LAP, lipid accumulation product; HOMA-IR, homeostasis model assessment of insulin resistance; 2h-PG, 2-hour oral postload glucose.

^*^According to ANOVA or Kruskal-Wallis test.

^§^All the groups significantly different (*P* < 0.05) to one another by post hoc comparisons of means.

^†^ *P* < 0.05 versus the other groups by post hoc comparisons of means.

^‡^ *P* < 0.05, 1st tertile versus 3rd tertile by post hoc comparisons of means.

**Table 2:** Features of female patients according to tertiles of waist circumference, LAP, and HOMA-IR.

	Age (years)^a^	BMI (kg/m^2^)^a^	Waist (cm)^a^	LAP (cm × mmol/L)^b^	Glucose (mmol/L)^b^	Insulin (*μ*U/dL)^b^	HOMA-IR^ b^	2h-PG (mmol/L)^b^
Overall (*N* = 294)	40.1 (12.1)	36.9 (6.2)	104.0 (13.0)	56.9 [40.3–89.1]	5.1 (0.6)	13.4 (7.8)	3.1 (2.0)	6.9 [5.8–8.4]
Waist tertiles								
I (*N* = 98)	37.6 (11.7)	31.9 (2.8)	90.8 (4.3)	38.7 [24.6–49.8]^†^	4.9 (0.6)^‡^	10.7 (4.5)	2.3 (1.1)^‡^	5.3 [6.1–7.4]^†^
II (*N* = 99)	41.4 (11.4)	36.3 (4.1)	102.2 (3.4)	58.6 [46.0–85.3]	5.1 (0.7)	13.1 (6.6)	3.0 (1.7)	7.2 [6.1–8.7]
III (*N* = 97)	41.3 (12.6)	42.6 (5.7)	119.3 (8.1)	90.2 [65.2–124.5]	5.2 (0.6)	16.5 (10.1)^†^	3.9 (2.5)	7.4 [6.3–8.8]
*P* value^*^	0.048	<0.001^§^	<0.001^§^	<0.001^§^	0.001	<0.001	<0.001^§^	<0.001
LAP tertiles								
I (*N* = 97)	38.3 (11.9)	33.4 (4.5)	94.9 (9.2)	32.2 [24.4–40.3]	4.8 (0.5)	10.4 (5.7)	2.3 (1.4)	6.2 [5.2–7.0]
II (*N* = 100)	39.5 (12.4)	36.7 (4.9)	103.2 (10.1)	59.6 [49.6–68.7]	5.0 (0.6)	13.0 (7.7)	2.9 (1.8)	6.5 [5.7–7.9]
III (*N* = 97)	42.4 (11.5)	40.8 (6.6)	114.1 (11.7)	105.0 [89.9–124.5]	5.3 (0.6)^†^	16.8 (8.4)	4.0 (2.2)	8.4 [7.3–10.5]^†^
*P* value^*^	0.058	<0.001^§^	<0.001^§^	<0.001^§^	<0.001	<0.001^§^	<0.001^§^	<0.001
HOMA tertiles								
I (*N* = 95)	40.1 (12.1)	35.0 (5.8)	100.3 (12.7)	44.3 [32.0–66.2]	4.7 (0.5)	6.4 (2.0)	1.3 (0.4)	6.3 [5.2–7.6]
II (*N* = 100)	39.4 (11.7)	36.8 (5.6)	103.3 (11.9)	56.9 [40.4–88.0]	5.0 (0.6)	12.1 (2.1)	2.7 (0.4)	6.9 [5.7–8.4]
III (*N* = 99)	40.7 (12.3)	38.9 (6.4)^†^	108.4 (13.0)^†^	83.0 [49.5–116.5]	5.4 (0.6)	21.7 (7.6)	5.2 (1.9)	7.7 [6.3–9.8]^†^
*P* value^*^	0.659	<0.001	<0.001	<0.001^§^	<0.001^§^	<0.001^§^	<0.001^§^	<0.001

Data are reported as mean (standard deviation)^a^ or median [interquartile range, 25th–75th percentile]^b^ or percentage. Percentages are calculated within groups.

BMI, body mass index; LAP, lipid accumulation product; HOMA-IR, homeostasis model assessment of insulin resistance; 2h-PG, 2-hour oral post load glucose.

^*^According to ANOVA or Kruskal-Wallis test.

^§^All the groups significantly different (*P* < 0.05) to one another by post hoc comparisons of means.

^†^ *P* < 0.05 versus the other groups by post hoc comparisons of means.

^‡^ *P* < 0.05, 1st tertile versus 3rd tertile by post hoc comparisons of means.

**Table 3:** Performance and comparison of insulin-resistance indices and waist circumference in identifying abnormalities in glucose metabolism by 2-hour postload glucose of an oral glucose tolerance test.

		LAP	HOMA-IR	Waist	*P* value^*^	*P* value^**^
IGT	OR (95% CI)^†^	2.37 [1.70–3.30]	1.39 [1.03–1.89]	1.44 [1.06–1.94]	0.006	0.978
	*P* value	<0.001	0.014	0.019		
	Cases correctly classified (%)	75.8	76.0	75.8		
	AUC (95% CI)	0.70 [0.65–0.75]	0.61 [0.56–0.66]	0.61 [0.56–0.66]		

T2-DM	OR (95% CI)^†^	3.17 [1.75–5.77]	3.12 [1.72–5.66]	1.33 [0.83–2.15]	0.920	0.024
	*P* value	<0.001	<0.001	0.235		
	Cases correctly classified (%)	91.9	91.7	91.9		
	AUC (95% CI)	0.77 [0.72–0.81]	0.76 [0.72–0.81]	0.66 [0.61–0.71]		

Composite IGT and T2-DM	OR (95% CI)^†^	3.12 [2.26–4.31]	1.90 [1.42–2.53]	1.51 [1.14–1.99]	0.007	0.185
	*P* value	<0.001	0.001	0.139		
	Cases correctly classified (%)	70.6	69.1	66.3		
	AUC (95% CI)	0.76 [0.71–0.80]	0.68 [0.63–0.73]	0.65 [0.59–0.69]		

LAP, lipid accumulation product; HOMA-IR, homeostasis model assessment of insulin resistance; OR, odds ratio; AUC, area under the curve; 95% CI, 95% confidence interval; IGT, impaired glucose tolerance; T2-DM, type-2 diabetes mellitus.

^*^LAP versus HOMA-IR.

^**^Waist circumference versus HOMA-IR.

^†^For linear increase over sex-specific tertiles of the distribution according to logistic regression adjusted for age (continuous) and smoking (current versus former smoker/nonsmoker).
